# Gene Silencing and Activation of Human Papillomavirus 18 Is Modulated by Sense Promoter Associated RNA in Bidirectionally Transcribed Long Control Region

**DOI:** 10.1371/journal.pone.0128416

**Published:** 2015-06-05

**Authors:** Muzaffer Ahmad Kassab, Madeeha Mudassir, Anand Singh, Muthuraman N, Mohita Bhagat, Jayanth Kumar Palanichamy, Pradeep Ramalingam, Kunzang Chosdol, Subrata Sinha, Parthaprasad Chattopadhyay

**Affiliations:** 1 Department of Biochemistry, All India Institute of Medical Sciences, Ansari Nagar, New Delhi, India; 2 Department of Pathology & Laboratory Medicine, David Geffen School of Medicine at University of California Los Angeles, Los Angeles, CA, United States of America; 3 Department of Biochemistry, Institute of Liver and Biliary Sciences, New Delhi, Vasant Kunj, India; 4 Weill Cornell Medical College, York Avenue, New York, NY, United States of America; 5 National Brain Research Center, Manesar, Gurgaon, Haryana, India; Wuhan University, CHINA

## Abstract

**Background:**

Recently various studies have demonstrated the role of promoter associated non-coding RNAs (pRNA) in dsRNA induced transcriptional gene silencing and activation. However the exact mechanistic details of these processes with respect to the orientation of pRNAs are poorly defined.

**Methodology/Principal Findings:**

We have identified novel sense and antisense long control region (LCR) associated RNAs (pRNAs) in HPV18 positive cervical cancer cell lines HeLa, C-4 I and C-4 II. Using dsRNAs against these pRNAs, we were able to achieve upregulation or downregulation of the sense and antisense pRNAs and the downstream E6 and E7 oncogenes. We present evidence that knockdown of the sense pRNA is associated with reduction in E6 and E7 oncogenes and an upregulation of antisense pRNA. Conversely upregulation of sense pRNA is accompanied by an induction of the oncogenes and a concomitant reduction in antisense pRNA. Moreover, the exact role of sense and antisense pRNAs in dsRNA mediated gene modulation was confirmed by their selective degradation using antisense phosphorothioate oligodeoxynucleotides (ODN). Degradation of sense pRNA with antisense ODN led to loss of dsRNA induced silencing and activation, suggesting that dsRNA mediated gene modulation requires sense pRNA. Both processes were accompanied with congruent changes in the methylation pattern of activating and repressive histones.

**Conclusion/Significance:**

Thus this data identifies and demonstrates the role of previously unknown important regulatory transcripts in HPV18 gene expression which can prove valuable targets in cervical cancer therapeutics. This mode of gene regulation by bidirectional transcription could be operational in other promoters as well and serve as a mechanism of regulating gene expression.

## Introduction

HPV induced cervical cancer is the world’s second most common gynecological cancer next only to breast cancer accounting for about 270, 000 deaths each year[1]. Among various HPV types, HPV16 and HPV18 are associated with 90% of cervical cancerous lesions[2,3]. Apart from cervical cancer, HPV is frequently detected in substantial proportion of other anogenital, head and neck, upper respiratory tract and even non‐melanoma skin cancers[4,5].

HPV encoded two oncogenes, E6 and E7, are the major driving factors for turning a normal epithelial cell into a cancerous cell[6–8]whose transcription is regulated by an upstream noncoding fragment of about 1000 bp known as LCR[9].

RNA interference (RNAi) has been used to degrade E6 and E7 mRNA (post transcriptional gene silencing or PTGS) of HPV successfully under both in vitro as well as in vivo conditions in HPV16 [10–12]and HPV18[13,14]integrated cells. However PTGS is transient in nature[15][15]and PTGS mediating siRNAs can lead to desensitization of cervical cancer cells to repeated treatment[12]. These problems can be overcome by targeting dsRNAs to the promoter region of genes to bring transcriptional gene silencing (TGS). Recently various reports have demonstrated that TGS and transcriptional gene activation (TGA) is mediated through transcripts present at the promoters known as promoter associated RNAs (pRNAs). pRNAs have been demonstrated as dsRNA targets for TGS and TGA[16–21]. TGS and TGA can lead to heritable changes in gene expression by changing methylation state of histones and/or DNA[22–24]. Our lab has demonstrated TGS in HIV1C and HPV16 utilizing the same mechanism[25,26].

We characterized the transcription profile of HPV18 LCR which led us to the identification of previously unknown transcripts in the LCR (pRNAs). LCR was found to be bidirectionally transcribed giving rise to sense and antisense pRNAs in various cell lines tested. dsRNA and ODNmediated alteration of pRNAs of different orientations at HPV18 LCR led to our finding that sense pRNA is a major regulator of E6 and E7 gene expression. We identified several dsRNAs against these pRNAs which can repress and activate transcription from HPV18 LCR. These newly identified transcripts may help us in further understanding of gene regulation in general and HPV18 gene regulation in particular.

## Materials and Methods

### Cell culture

C-4 II was procured from ATCC (American Type Culture Collection) USA while C-4 I[27]was a kind gift from Pulivarthi Rao (Baylor College of Medicine, Tx. USA). HeLa cells were obtained from NCCS (National Centre for Cell Sciences) India. HeLa, C-4 I(27) and C-4 II cells are HPV18 positive cell lines. HeLa has about 10 to 50 copies of HPV18 while C-4 I and C-4 II contain one copy of HPV18 each. HeLa cells were maintained in DMEM (Sigma Corporation St. Louis, MO) while C-4 I and C-4 II cells were grown in Waymouth's MB 752⁄1 (Life Technologies Carlsbad, CA). The basic media was supplemented with 10% heat inactivated FCS (Life Technologies) and cells were grown under 5% CO_2_ at 37°C.

### dsRNA and ODN transfection

The dsRNAs (S1 Table) were generated against three different regions of pRNA, three to four dsRNAs were designed against each region with each dsRNA shifted in position by two nucleotides in each group. Group one included S1, S2 and S3 dsRNAs (-7448 to -7470), group two included S4, S5, S6 and S7 dsRNAs (-7567 to -7591), group three contained S8, S9 and S10 dsRNAs (-7598 to -7620) and a control dsRNA was also designed.

For transfection, initial plating density of cells was 10^5^ cells per well in a six-well plate, 3 × 10^5^ cells per 25 cm^2^ flask and 10^6^ cells per 75 cm^2^ flask. Twenty four hours later transfection was carried out with dsRNAs (Eurofins, MWG Germany) or ODNs (IDT, USA) at 100nM concentration using OligofectamineTransfection Reagent (Life Technologies) as per the manufacturer's protocol. Transfected cells were maintained for 72 hours unless otherwise stated.

ODN treatment plus dsRNA transfection was carried out by transfecting cells with the S1, S5 or S9 dsRNAs 24 hours later after respective ODNs treatment (100 nM, S1 Table). RNA was isolated from the cells 72 hours post 2^nd^ transfection.

### RNA analysis

RNA was isolated from the cells using TRI Reagent (Sigma-Aldrich) and DNase treated (Thermo Fisher Scientific, USA) according to the manufacturer’s guidelines. RNA (250 ng-1.5 μg) was reverse transcribed (RT) using MMLV Reverse Transcriptase (Thermo Fisher Scientific, USA) using random decamers except in the case of directional-RT (sense or antisense pRNA) in which a gene specific primer (100 pM) was used.

Real-time RT-PCR (qRT-PCR) was done on Rotor-Gene 6000 real-time PCR machine (Corbett Research, Australia) using indicated gene specific primers (S2 Table). Multiple reference genes i.e. 18S, POLR2A, PPIA, and β-actin were used for accurate quantification of gene expression using the Relative Expression Software Tool which is available online at http://www.genequantification.de/rest.html. This software applies a mathematic model that takes into account the variable PCR efficiencies of the target gene and reference genes. Thus using multiple reference genes improves the reliability of the assay than using the conventional single reference gene normalization method[28].

### pRNA analysis

For the detection of pRNAs, total RNA was isolated from HeLa, C-4 I and C-4 II cells using TRI Reagent (Sigma-Aldrich) and DNase treated (DNase I, Thermo Fisher Scientific, USA) according to the manufacturer’s guidelines. The cDNA was then PCR amplified with P1, P2, P3 and P4 primers. The PCR products obtained were electrophoresed on 1% agarose (Pronadisa, Spain) gel in 1 X TAE buffer at 4°C using a constant voltage of 5–10 V/cm. The gel was stained with ethidium bromide and the resolved DNA bands were visualized under UV using transilluminator (AlphaImagerHP, Fisher Scientific). The PCR products were gel purified by Wizard SV Gel and PCR Clean-Up System (Promega, USA) strictly following the manufacturer’s protocol and commercially sequenced from Xcelris Genomics (India).

In order to determine the orientation, contiguity and expression of pRNAs, total RNA was reverse transcribed (Thermo Fisher Scientific) using indicated RNA specific (100 pM) forward (antisense specific) or reverse (sense specific) primers. The cDNA was PCR amplified with both the forward and reverse primers together. During directional qRT-PCR, data was first normalized to internal 18S levels and then expressed as fractions of control values.

### Bisulfite conversion

DNA was isolated from dsRNA transfected cells using Gentra Puregene Blood kit (Qiagen USA) and 200 ng DNA was bisulfite modified with EpiTect Bisulfite kit (Qiagen) according to the manufacturer’s guidelines. The software available at http://bisearch.enzim.hu/ was used for the designing of both outer and nested primers (S3 Table) and the PCR products obtained were commercially sequenced from Xcelris Genomics (India).

### Chromatin immunoprecipitation (ChIP) assay

ChIP assay for H3K9me2 (Abcam #mAbcam1220), H3K27me3 (Abcam #mAbcam6002) and H3K4me2 (Abcam #ab32356) tails was performed using EZ-ChIP Kit(Millipore USA) according to manufacturer’s instructions. Immunoprecipitated DNA was analyzed by PCR for 25–30 cycles. PCR primers used for ChIP analysis are described in S2 Table.

### *Micrococcal nuclease* (MNase) assay

MNase accessibility assay was carried out as described previously [29]. dsRNA and ODN treated cells (2 x10^5^) were scraped from 25 cm^2^ culture flask and pelleted down by centrifugation at 2000 rpm for 10 min at 4°C. Cell pellet was washed twice in ice-cold PBS and resuspended in 200 mL ice-cold NP40 lysis buffer (10 mM Tris-Cl pH 7.4, 10 mM NaCl, 3 mM MgCl2, 0.5% NP40, 0.5 mM spermidine, 0.15 mM spermine) followed by incubation on ice for 5 min. The nuclei were pelleted down at 3000 rpm for 5 min at 4°C and resuspended in 200 mL of MNase digestion buffer (100 mM Tris-HCl pH 7.4, 3 M NaCl, 15 mM spermine, 50 mM spermidine, 0.6 M KCl, and 100 mM CaCl_2_). 100 μl of suspension was digested with 2U of MNase for 10 min at 37°C and remaining 100 μl was kept undigested. MNase reaction was stopped by addition of EDTA (5 mM final concentration) at 65°C for 10 minutes. DNA from both digested and undigested nuclei was isolated by Gentra Puregene Blood kit (Qiagen) and analyzed by real-time PCR. The ratio of undigested (UD) to digested (D) DNA of gene of interest (GOI) in each sample was calculated using the Delta Delta Ct method e.g. ratio of undigested to digested DNA = 2^ ^[Ct value of CHR16 (UD)—Ct value of GOI (UD)]^/2^ ^[Ct value of CHR16 (D)—Ct value of GOI (D)]^. The primers amplifying a fragment of centromere region of chromosome 16 was used as a reference gene for the analysis as it is maintained in heterochromatized form in the cells. The primers used for MNase analysis are described in S2 Table.

### Trichostatin A (TSA) and 5-Azathymidine (AZA) treatment

Roughly 2 x10^5^ cells were transfected with S1, S5 or S9 dsRNAs (100 nM) and 24 hours later treated with TSA (400 nM), AZA (5 μM) or DMSO. RNA was isolated from the cells 48 hours after the treatment and analyzed by real-time PCR.

### Statistical analyses

All real-time PCR experiments involving pRNA, E6 and E7 were performed in triplicate and repeated at least three times while MNase and ChIP assay experiments were repeated at least twice. The data are presented as the mean ± SD. Significance in the differences was determined using Student’s t-test. P values less than 0.05 were taken as significant.

## Results

### Identification and characterization of LCR associated RNA (pRNA) in HPV18 LCR

**Fig 1:**
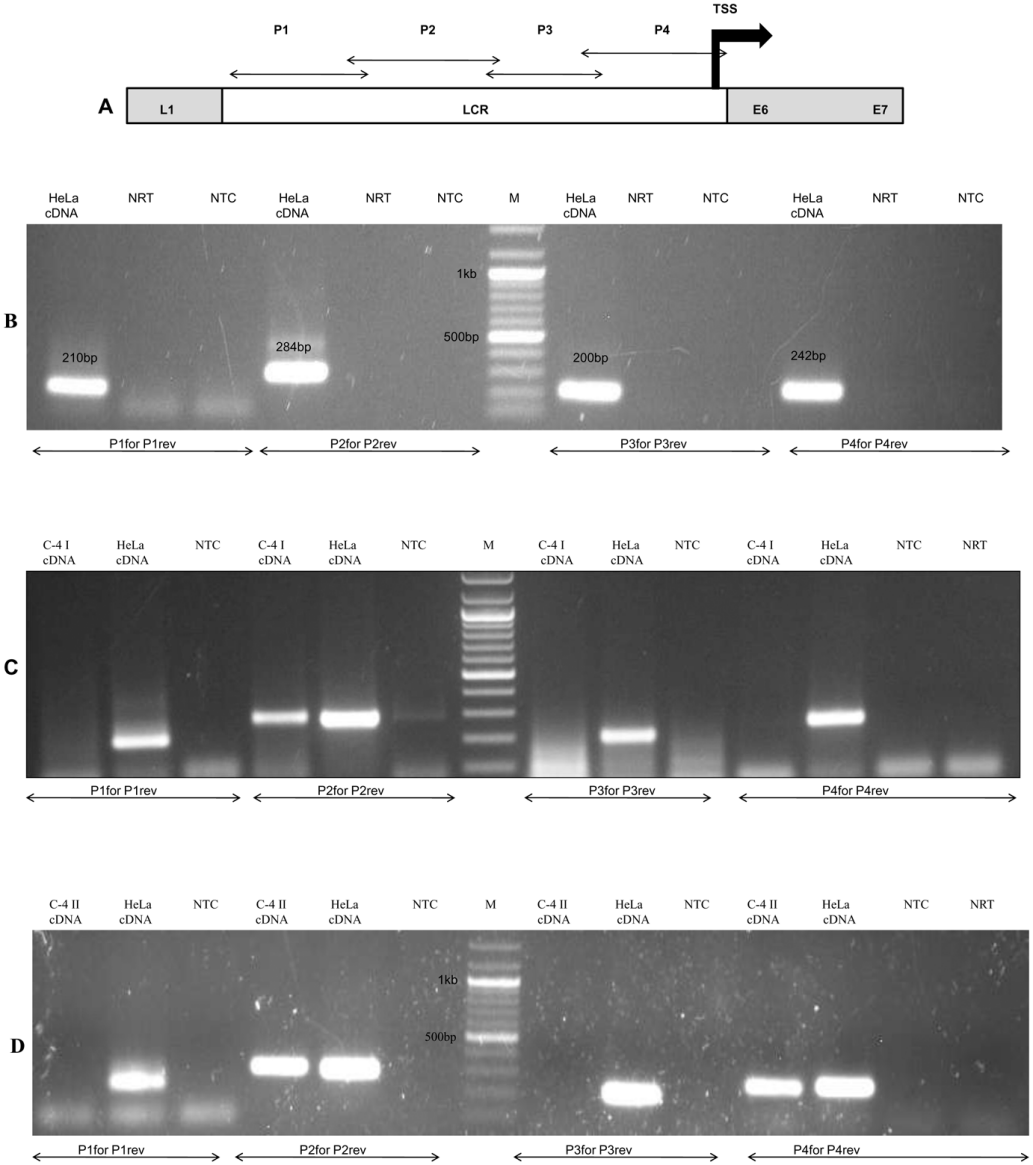
Detection of pRNA at the LCR of HPV18 positive cell lines. (A) Schematic representation of HPV18 LCR showing the position of four LCR specific primers. The flanking genes and the transcription start site (TSS) are also depicted. For the detection of pRNA across LCR, RNA was isolated from HeLa, C-4I and C4-II cells and subjected to RT-PCR using four sets of LCR specific overlapping primers P1, P2, P3 and P4. (B) Gel picture showing pRNA distribution across HeLa LCR. pRNA found at all the regions of LCR (C) pRNA presense at C-4 I LCR. pRNA was detected at P2 region only. (D) pRNA presense at C-4 II LCR. pRNA was detected at P2 and P4 regions of LCR. P1for P1rev: PCR performed with P1F and P1R primers, P2for P2rev: PCR performed with P2F and P2R primers, P3for P3rev: PCR performed with P3F and P3R primers, P4for P4rev: PCR performed with P4F and P4R primers. HeLa cDNA: HeLa cDNA used as positive PCR Control, NRT:negative RT, NTC: no template Control, M: 100bp ladder.

RNA was isolated from HeLa, C-4I and C-4 II cells and subjected to RT-PCR using four pairs of LCR specific primers overlapping regions P1, P2, P3 and P4 (Fig 1A) to determine the transcription of pRNA across HPV18 LCR. In HeLa cells, all the primers led to the amplification of the region of desired size (Fig 1B) while in C-4 I pRNA was detected only at P2 region (Fig 1C) and in C-4 II cells pRNAs were detected only at P2 and P4 regions (Fig 1D). Sequencing of the PCR bands confirmed their sequence similarities with the LCR at the respective regions.

**Fig 2:**
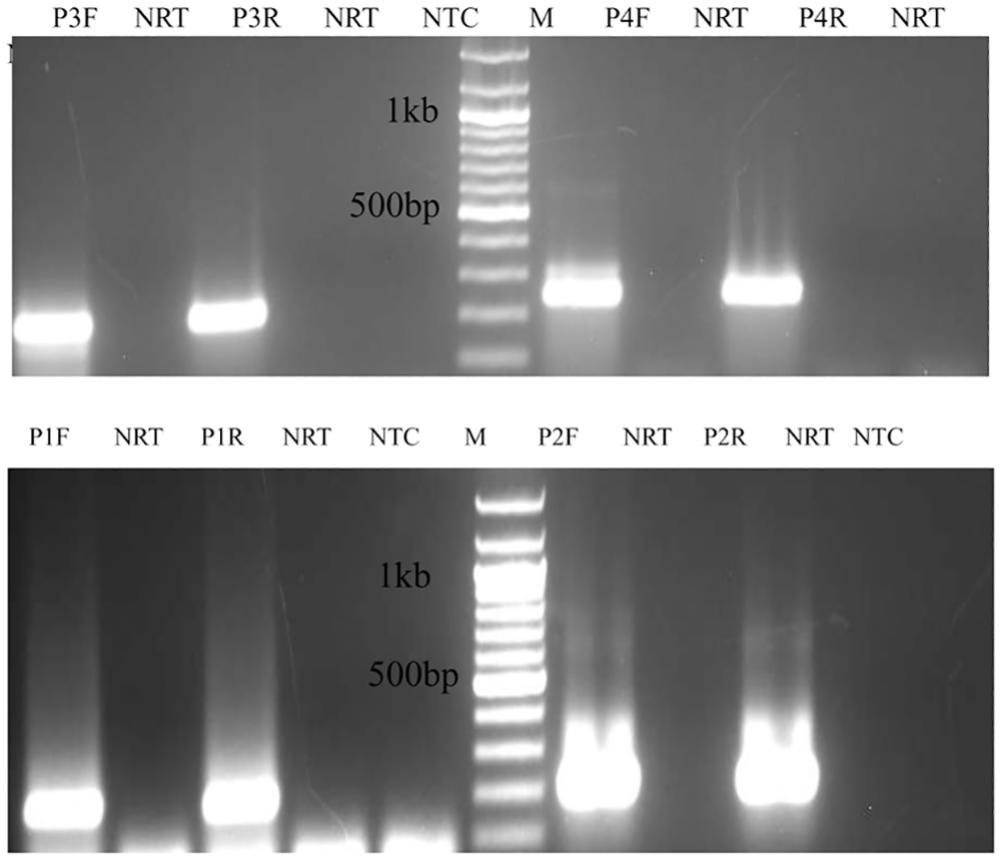
Determination of pRNA orientation in HPV18 integrated HeLa cells. RNA extracted from the cells was DNase treated and subjected to reverse transcription with pRNA specific forward (antisense specific) or reverse (sense specific) primers and PCR performed with forward and reverse primers together. P1F: RT performed with P1F primer and PCR with P1F and P1R primers, P1R: RT performed with P1R primer and PCR with P1F and P1R primers, P2F: RT performed with P2F primer and PCR with P2F and P2R primers, P2R: RT performed with P2R primer and PCR with P2F and P2R primers, P3F: RT performed with P3F primer and PCR with P3F and P3R primers, P3R: RT performed with P3R primer and PCR with P3F and P3R primers, P4F: RT performed with P4F primer and PCR with P4F and P4R primers, P4R: RT performed with P4R primer and PCR with P4F and P4R primers, NRT:negative RT, NTC: no template Control, M: 100bp ladder.

We next sought to determine the orientation of pRNA with respect to E6 and E7 mRNA. Same set of four primers were used for performing directional RT-PCR. In HeLa cells primer specific bands were formed with all the forward and reverse primers (Fig 2) suggesting that pRNA is present in both sense and antisense orientations at these regions. On performing similar set of experiments in C-4 I and C-4 II cells we found pRNA to be bidirectionally transcribed at P2 region in both the cell lines (Figures A and B in S1 File) which were named as *C-4 I LCR sense pRNA* and *C-4 I LCR antisense pRNA* for C-4 I and *C-4 II LCR sense 1 pRNA and C-4 II LCR antisense 1 pRNA* for C-4 II. Similarly pRNA was also found to be bidirectionally transcribed at P4 region only in C-4 II LCR designated as *C-4 II LCR sense 2 pRNA* and *C-4 II LCR antisense 2 pRNA* (Figure B in S1 File). The PCR products upon sequencing confirmed their sequence match with the LCR at the respective regions.

**Fig 3:**
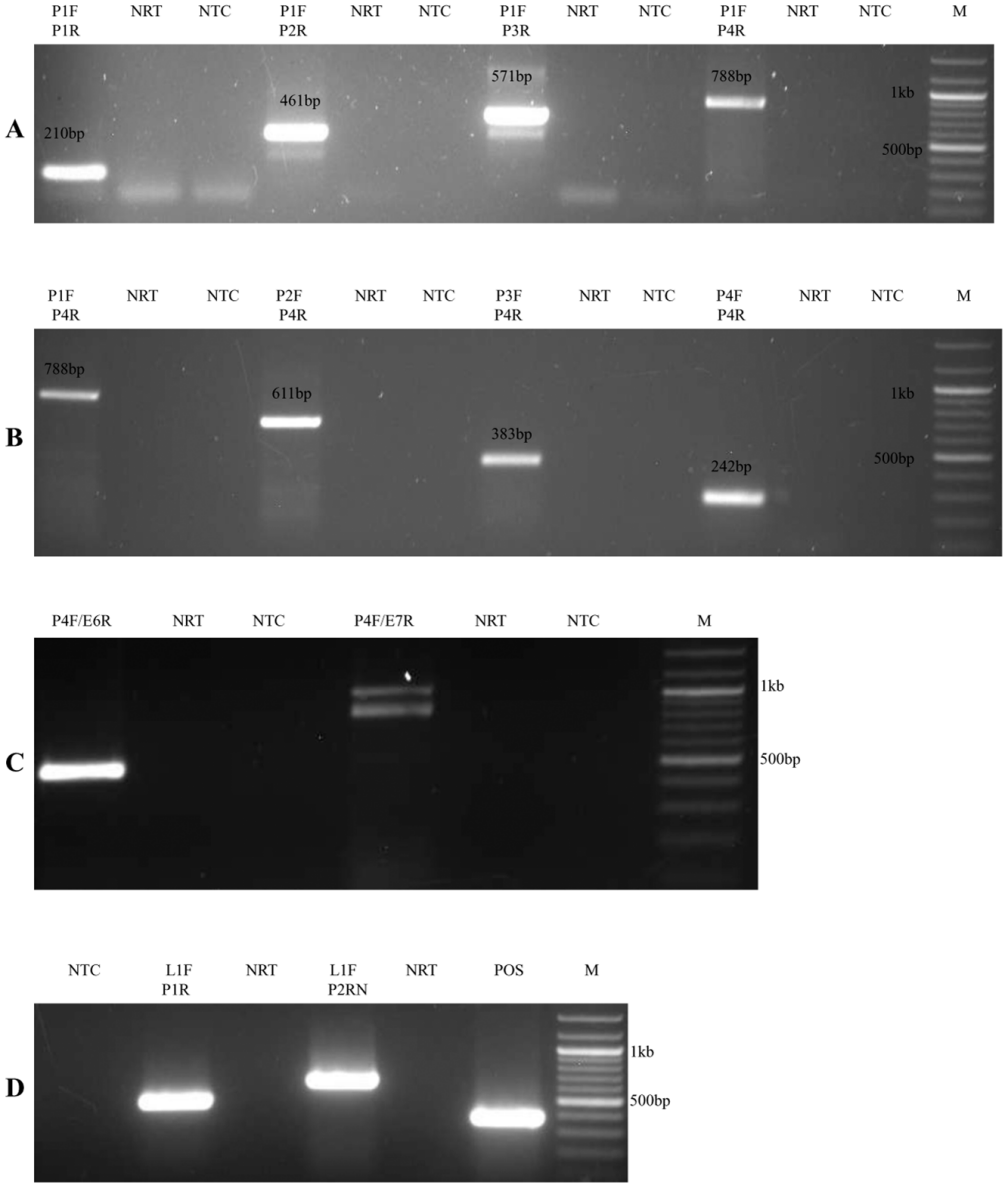
Sense and antisense pRNA extend throughout LCR and into the flanking genes in HeLa cells. (A) Antisense pRNA: RNA was reverse transcribed using P1F primer (antisense pRNA specific). The cDNA formed was PCR amplified using different primers combinations i.e. P1F and P4R, P2F and P4R, P3F and P4R, P4F and P4R. (B) Sense pRNA: P1R primer (sense pRNA specific) was used for reverse transcribing RNA and the cDNA formed was amplified using indicated primers i.e. P1F and P1R, P1F and P2R, P1F and P3R, P1F and P4R. (C) Sense pRNA extends into E6 and E7 coding regions. P1F primer was used for reverse transcribing HeLa RNA and the cDNA formed was PCR amplified with different primers as indicated, P4F/E6R: PCR with P4 forward and E6 reverse primers, P4F/E7R: PCR with P4 forward and E7 reverse primers (D) Antisense pRNA extends into L1 coding region. P4R primer was used for reverse transcribing HeLa RNA and the cDNA formed was PCR amplified with different primers asindicated, L1F/P1R: PCR with L1 forward and P1 reverse primers, L1F/P2RN: PCR with L1 forward and P2RN primers, NRT:negative RT, NTC: no template Control, M: 100bp ladder.

Next we tested the contiguity of the pRNA in the HeLa LCR to determine if the pRNAs are extending throughout the LCR or confined as discrete transcripts to each region. In order to determine the contiguity, RNA was isolated from HeLa cells and reverse transcribed with P1F (antisense pRNA P1 regionspecific) and P4R (sense pRNA P4 regionspecific) primers separately. The cDNAs were PCR amplified using indicated primers (Fig 3A and 3B). We obtained pRNA specific PCR bands with all the primer combinations which were confirmed by sequencing. The sense and antisense pRNAs were named *Hela LCR sense pRNA* and *HeLa LCR antisense pRNA* respectively. In addition sense pRNA extended into the E6 and E7 loci and the antisense pRNA extended into the L1 locus as determined by directional RT-PCR using specific primers (Fig 3C and 3D).

### Designing and screening of dsRNA against pRNA

**Fig 4:**
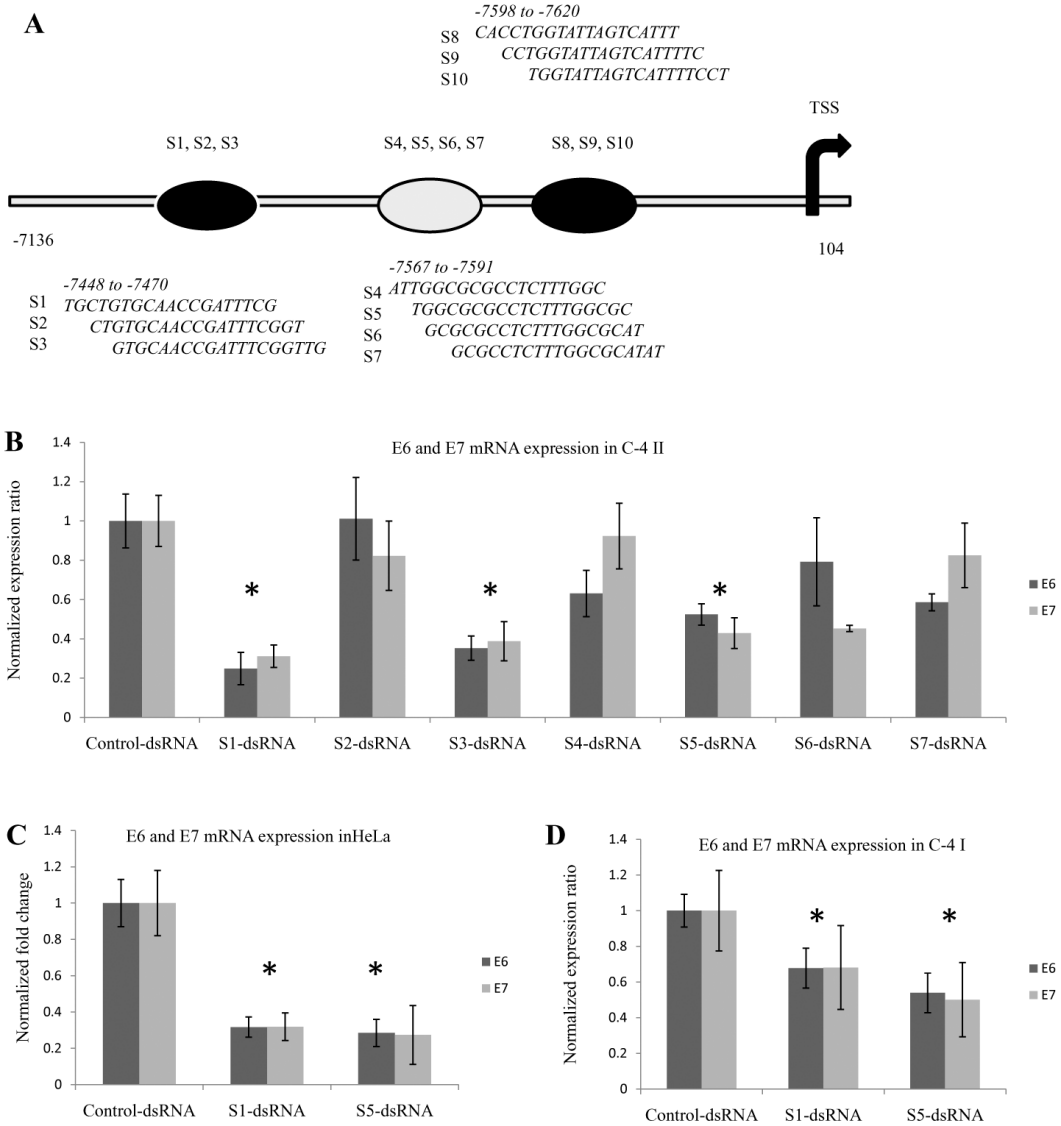
Screening of dsRNAs targeting HPV18 pRNAs. (A)Schematic representation of HPV18 LCR and the position of different dsRNA target sites. (B) C-4 II cells transfected with seven dsRNAs (100nM). C) S1 and S5 dsRNA transfection in HeLa cells (D) C-4 I cells transfected with S1 and S5 dsRNAs. Cells were transfected with the respective dsRNA and RNA was isolated 72 hours post transfection and analyzed by Real-Time RT-PCR. Expression ratio was calculated with respect to 18S rRNA, POLR2A, PPIA and β-actin by REST software and normalized to that of Control dsRNA. E6: Expression of E6 oncogene upon dsRNA transfection. E7: Expression of E7 oncogene upon dsRNA transfection. Control-dsRNA: Control dsRNA transfected cells, (*) P < 0.05.

**Fig 5:**
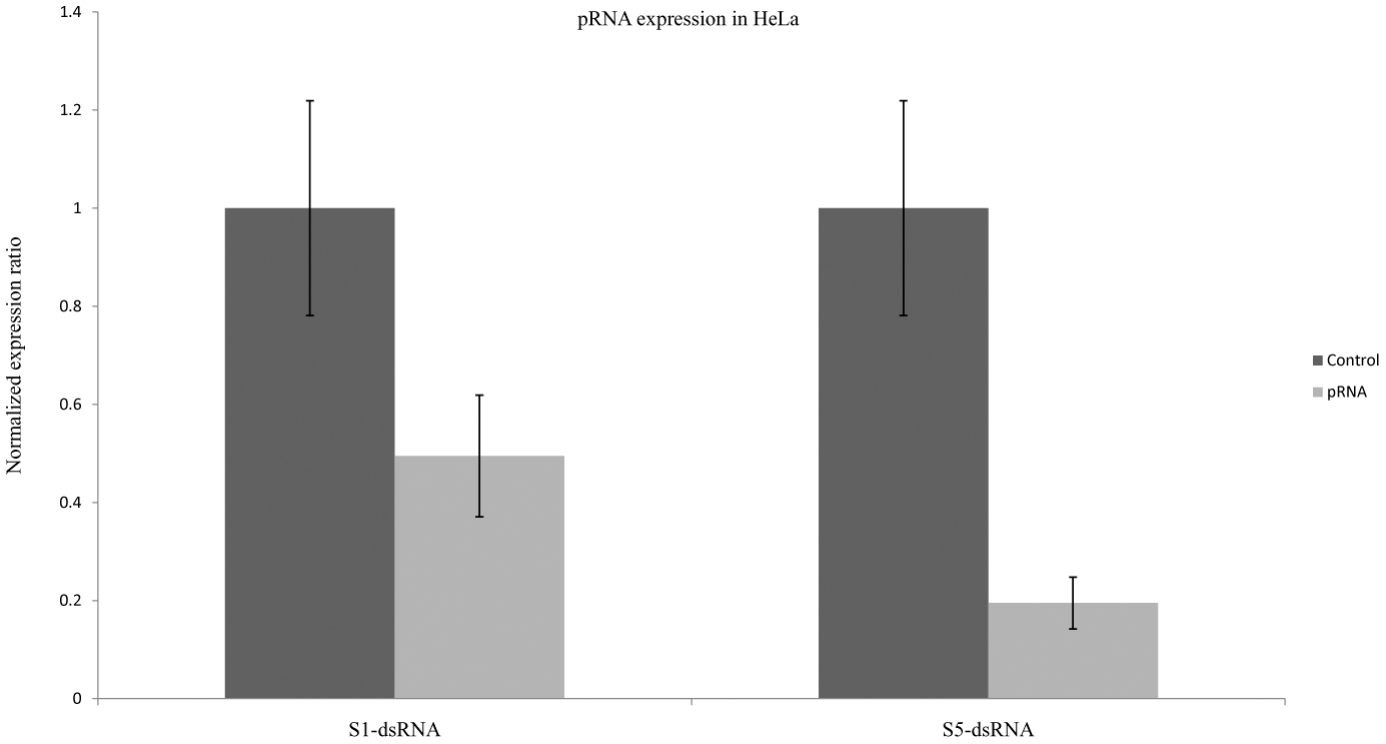
pRNA expression in HeLa cells. Cells were transfected with S1 or S5 dsRNAs (100nM) and analyzed for pRNA expression 72 hours post transfection. Analysis was done with Real Time PCR using pRNA specific primers. S1 and S5 transfection showed significant (p < 0.01) decrease in pRNA expression. Expression ratio was calculated with respect to 18S, POLR2A, PPIA and β-actin by REST software. pRNA: pRNA targeting S1 or S5 dsRNAs transfected cells. Control-dsRNA: Control dsRNA treated cells.

Although there was variation in the size and distribution of pRNA among different HPV18 cell lines, it was present at P2 region of LCR in all the three cell lines. To determine the function of pRNA,we designed seven (S1-S7) pRNA specific complementary dsRNAs (Fig 4A) targeting two different regions of pRNA in the P2 region. Each dsRNA contained at least two CpG sites in the target region. dsRNAs targeting CpG sites have been associated with TGS[22,30]. These dsRNAs were screened in C-4 II cells and E6 and E7 gene expression was analyzed using specific primers by Real Time PCR (qRT-PCR) 72 hours after transfection (Fig 4B). Among the seven dsRNAs used only S1, S3 and S5 caused significant fall in E6 and E7 expression by around 73%, 60% and 55% respectively in comparison to control dsRNA treated cells. On transfecting one dsRNA from each region (S1 and S5) in HeLa(Fig 4C) and C-4 I (Fig 4D) cells similar knockdown of E6 and E7 expression was observed. Moreover, along with E6 and E7 mRNA, total pRNA (both sense and antisense) was also significantly reduced (P < 0.01) in all transfected cells types (Fig 5A, Figures A-B in S2 File).

### Effect of dsRNA transfection on bidirectional pRNA transcription

RNA from S1 and S5 transfected cells was analyzed by directional qRT-PCR to find the effect of dsRNA transfection on the expression of sense and antisense pRNAs in HPV18 positive cells.

**Fig 6:**
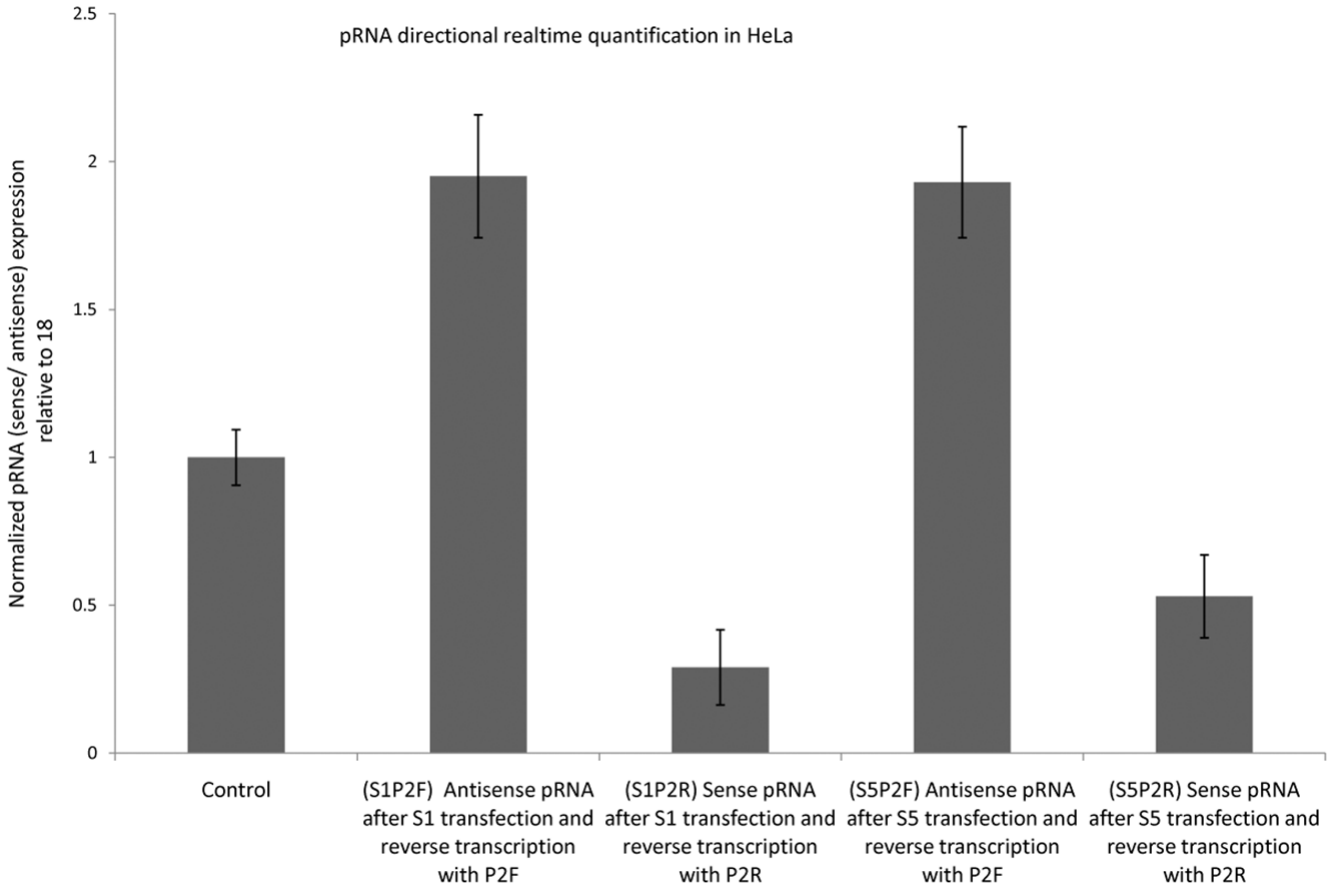
Change in bidirectional pRNA transcription in HeLa after S1 or S5 dsRNA transfection. The levels of sense and antisense pRNAs were checked using directional qRT-PCR using P2 primer. Fold changes in sense and antisense pRNA expression were calculated using delta-delta-Ct method with respect to 18S as internal Control. Control: Control dsRNA treated cells, S1P2F: cells transfected with S1 dsRNA followed by RNA isolation and reverse transcription with P2F primer, S1P2R: cells transfected with S1 dsRNA and RNA isolated was reverse transcribed with P2R primer, S5P2F: cells transfected with S5 dsRNA and RNA isolated was reverse transcribed with P2F primer, S5P2R: cells transfected with S5 dsRNA and RNA isolated was reverse transcribed with P2R primer.

Sense pRNA, which is in the same orientation as E6 and E7 mRNA, was subjected to directional reverse transcription using P2R primer and the cDNA obtained was analyzed using q-PCR with P2F and P2R primers together. Antisense pRNA (with respect to mRNA) was reverse transcribed using P2F primer and the cDNA was analyzed using q-PCR with P2F and P2R primers. There was significant decrease (p *<*0.002) in sense pRNA expression and a concomitant increase (p *< 0*.*01)* in the level of antisense pRNA after S1 and S5 dsRNA transfection in HeLa and C-4 II cells (Fig 6 and S1 Fig).

### pRNA mediated E6 and E7 gene activation

**Fig 7:**
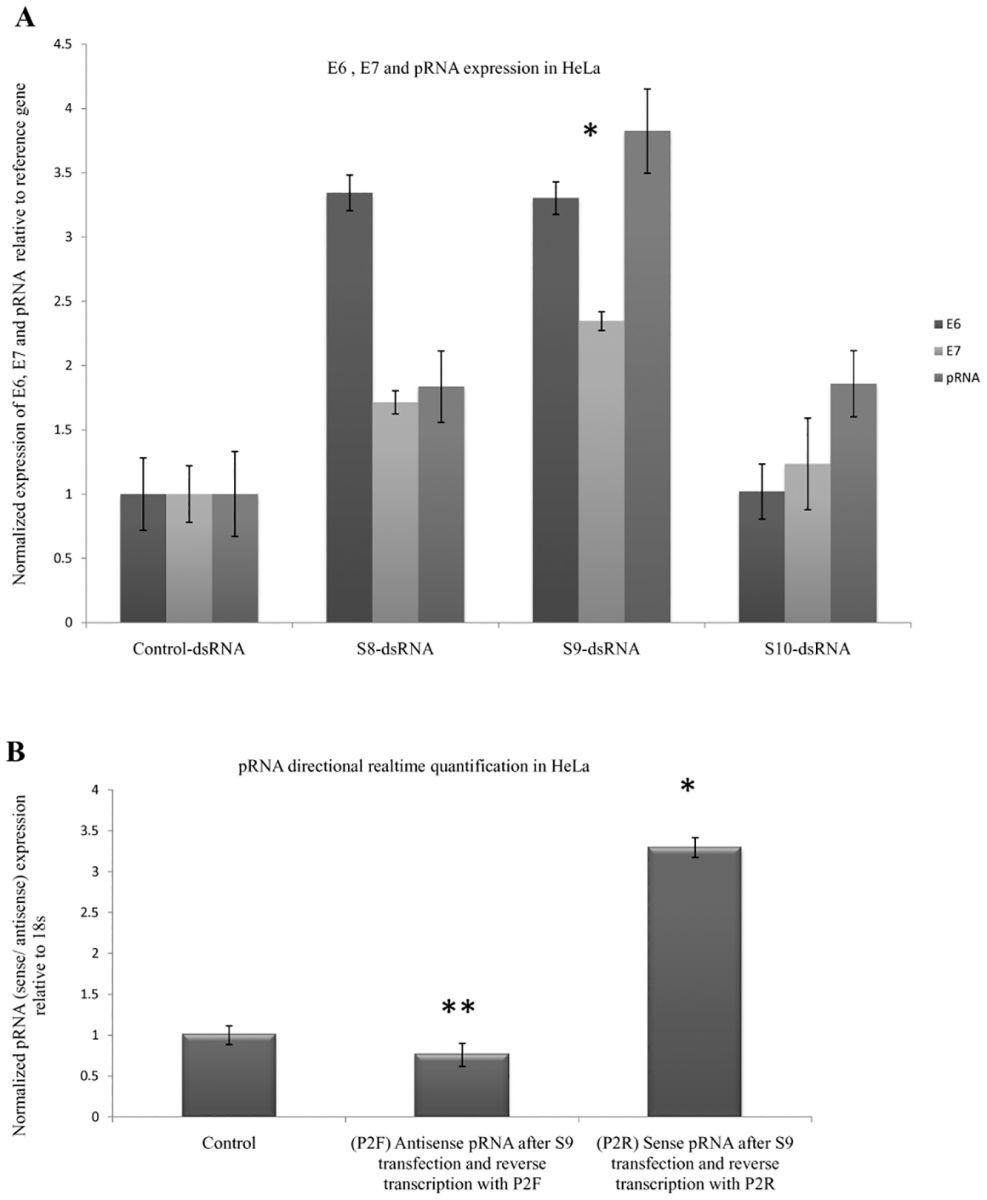
pRNA mediated gene activation. (A) Screening of three dsRNAs (100nM) targeting non-CpG sites in the pRNA of HPV18 in HeLa cells. RNA from the transfected cells was analyzed by Real time PCR 72 hours post transfection. Expression ratio was calculated with respect to 18S, POLR2A, PPIA andβ-actin by REST software. S8-dsRNA: S8 dsRNA transfected cells, S9-dsRNA: S9 dsRNA transfected cells, S10-dsRNA: S10 dsRNA transfected cells. Control: Control dsRNA transfected cells. (*) P < 0.01, (**) P = 0.03. (B) Change in bidirectional transcription after S9 dsRNA transfection. The levels of sense and antisense pRNAs were checked by transfecting Hela cells with S9 dsRNA followed by RNA isolation 72 hours post transfection. The cDNA was then analyzed by Real-Time PCR using P2 primer. P2F: cells transfected with S9 dsRNA followed by RNA isolation and reverse transcribed with P2F primer which reverse transcribes antisense pRNA, P2R: cells transfected with S9 dsRNA followed by RNA isolation and reverse transcription with P2R primer which reverse transcribes sense pRNA. Expression ratio was calculated with respect to 18S rRNA using delta-delta Ct method normalized to Control dsRNA treated samples.

Amongst the seven CpG containing dsRNAs used, S1 and S5 decreased pRNA, E6 and E7 expression while others had no significant effect on the expression of these RNAs. Recent literature suggests that non-CpG targeting dsRNAs are associated with induction of the gene expression[19]. So, we designed and screened another group of three dsRNAs (S8, S9 and S10) targeting non-CpG sites in the pRNA. S9 dsRNA was associated with induction of pRNA (2 fold), E6 (2.5 fold) and E7 (3.5 fold) 72 hours post transfection while control cells remained unaffected as determined by qRT-PCR in HeLa cells (Fig 7A).

RNA from S9 transfected HeLa cells was assessed by bidirectional qRT-PCR to determine the effect of non-CpG targeting dsRNA on bidirectional pRNA expression (Fig 7B). Transfection with S9 dsRNA was associated with two fold increase in the expression of sense pRNA and a concomitant decrease in the level of antisense pRNA.

### pRNA activation and repression is associated with change in epigenetic modifications

**Fig 8:**
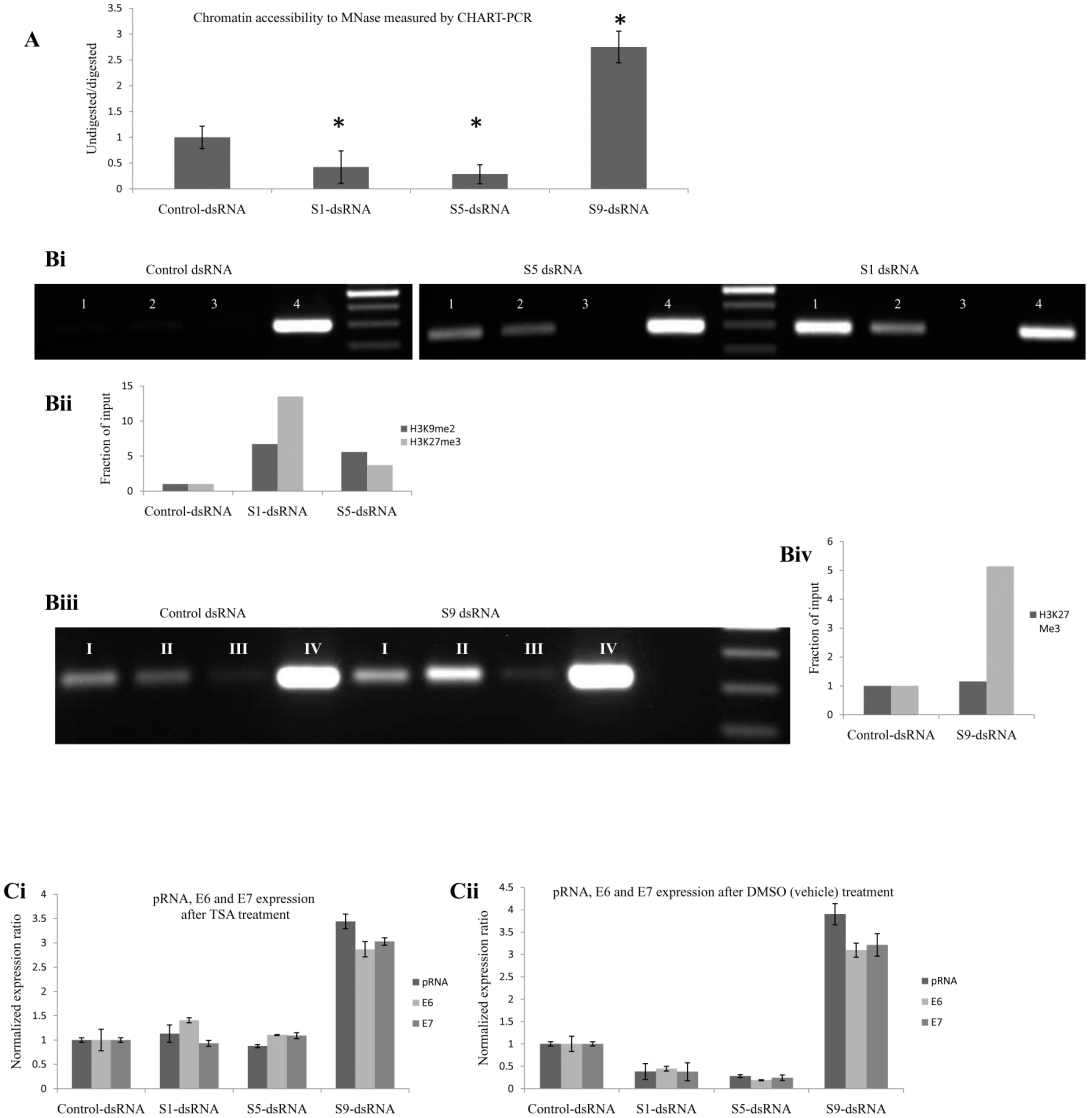
Epigenetic modifications post dsRNA transfection. (A)Chromatin remodeling after S1, S5 or S9 dsRNA transfection. Significant reduction in MNase digestion 72 hours after S1 and S5 transfection while increase in the accessibilty after S9 transfection in HeLa cells. Cells were transfected with the respective dsRNAs followed by MNase-PCR 72 hours post transfection. S1:S1 dsRNA transfected cells, S5-dsRNA: S5 dsRNA transfected cells, S9-dsRNA: S9 dsRNA transfected cells, Control-dsRNA: Control dsRNA transfected cells (*) P < 0.01. Bi-Biv. Enrichment of H3K9me2, H3K27me3 and H3K4me2 at dsRNA target sites. dsRNA induced TGS is associated with increase in H3K9me2 and H3K27me3 while TGA is associated with gain in H3K4me2. HeLa cells were transfected with respective dsRNAs followed by ChIP analysis 72 hours later using the desired antibodies to pull the target DNA. Precipitated DNA was analyzed by PCR using region specific primers. DNA pulled down in the absence of antibody (mouse IgG) served to identify background amplification while input DNA was amplified as a loading Control. (Bi) Gel electrophoretogram showing PCR products obtained after ChIP in S1 and S5 dsRNA transfected cells. (Biii) Gel electrophoretogram showing PCR products obtained after ChIP in S9 dsRNA transfected cells. (Bii and Biv) Densitometric analysis of ChIP data shown in A and B, obtained with ImageJ software. Relative enrichment = ChIP/Input DNA. 1: H3K9me2 immunoprecipitated PCR, 2 and I: H3K27me3 immunoprecipitated PCR, 3 and III: mouse IgG immunoprecipitated PCR, 4 and IV: Input DNA, II: H3K4me2 immunoprecipitated PCR. Ci-Cii. Effect of HDAC inhibition on TGS and TGA. Expression ratio of pRNA, E6 and E7 after S1, S5 or S9 transfection. HeLa cells were transfected with dsRNA (100nM) and 24 hours later treated with DMSO or TSA (400nM) followed by RNA isolation 48 hours after the treatment. (Ci) TSA treatment in dsRNA transfected cells. (Cii) DMSO (solvent for TSA and AZA) treatment in dsRNA transfected cells.

Our findings suggest that TGS and TGA of HPV18 are associated with sense pRNA levels in a bidirectionally transcribed LCR with decrease and increase in sense pRNA levels resulting in suppression and activation respectively. To elucidate if the dsRNA mediated pRNA modulation is associated with change in chromatin status, MNase assay was carried out in HeLa cells post S1, S5 or S9 transfection. In S1 and S5 dsRNA treated cells, there was reduction in accessibility (55% and 70% respectively) of the target region to MNase in comparison to control dsRNA treated cells. In contrast, when HeLa cells were transfected with S9 dsRNA, the target region became more sensitive to MNase digestion, signifying increased accessibility, while there was no change in the accessibility of control dsRNA transfected cells (Fig 8A).

Now to elucidate the mechanism behind the observed changes in chromatin accessibility, chromatin immunoprecipitation (ChIP) assay was carried out against activating and repressive histone methylation markers. S1 transfection in HeLa cells led to H3K9me2 and H3K27me3 enrichment at the target site by 6.7 and 13.5 fold respectively while S5 transfection increased H3K9me2 and H3K27me3 by 5.5 and 3.7 fold respectively (Fig 8Bi and 8Bii). In contrast, cells transfected with S9 dsRNA showed no change in the enrichment of H3K27me3 while activating histone marker H3K4me2 increased 5 fold at the region (Fig 8Biii and 8Biv).

When the cells were treated with HDAC inhibitor TSA, there was abrogation (*P > 0*.*05)* of S1 and S5 mediated TGS indicating that the HDAC inhibitor TSA reverted back dsRNA mediated heterochromatization while there was no change in S9 mediated TGA (*P < 0*.*01)* (Fig 8Ci). dsRNA mediated TGS and TGA were unaffected (*P < 0*.*01)* when the cells were treated with DMSO which is a solvent for TSA (Fig 8Cii). This indicates that heterochromatization of target region by HDAC mediated deacetylation is one of the processes involved in TGS.

There was no change in the methylation of the target DNA or the adjacent region as found by bisulphite sequencing (Figures A-E in S3 File). Moreover S1 and S5 mediated pRNA, E6 and E7 downregulation was unaffected (p < 0.01) with AZA (inhibitor of DNA methyltransferases) treatment (Figure F in S3 File) which confirms absence of DNA methylation during TGS. This is in accordance with various previous reports of TGS occurring in the absence of DNA methylation.

### Sense and antisense pRNA requirement in E6 and E7 expression

Recently various studies have shown that promoter associated transcripts are the direct targets of dsRNAs involved in TGS[16,17]. We synthesized three ODNs, one (antisense ODN) targeting the sense pRNA, another (sense ODN) against the antisense pRNA and a control ODN to determine the requirement of pRNA in E6 and E7 mRNA expression. ODNs after hybridization to a complementary RNA induce degradation of the RNA strand by RNase H pathway[31].

**Fig 9:**
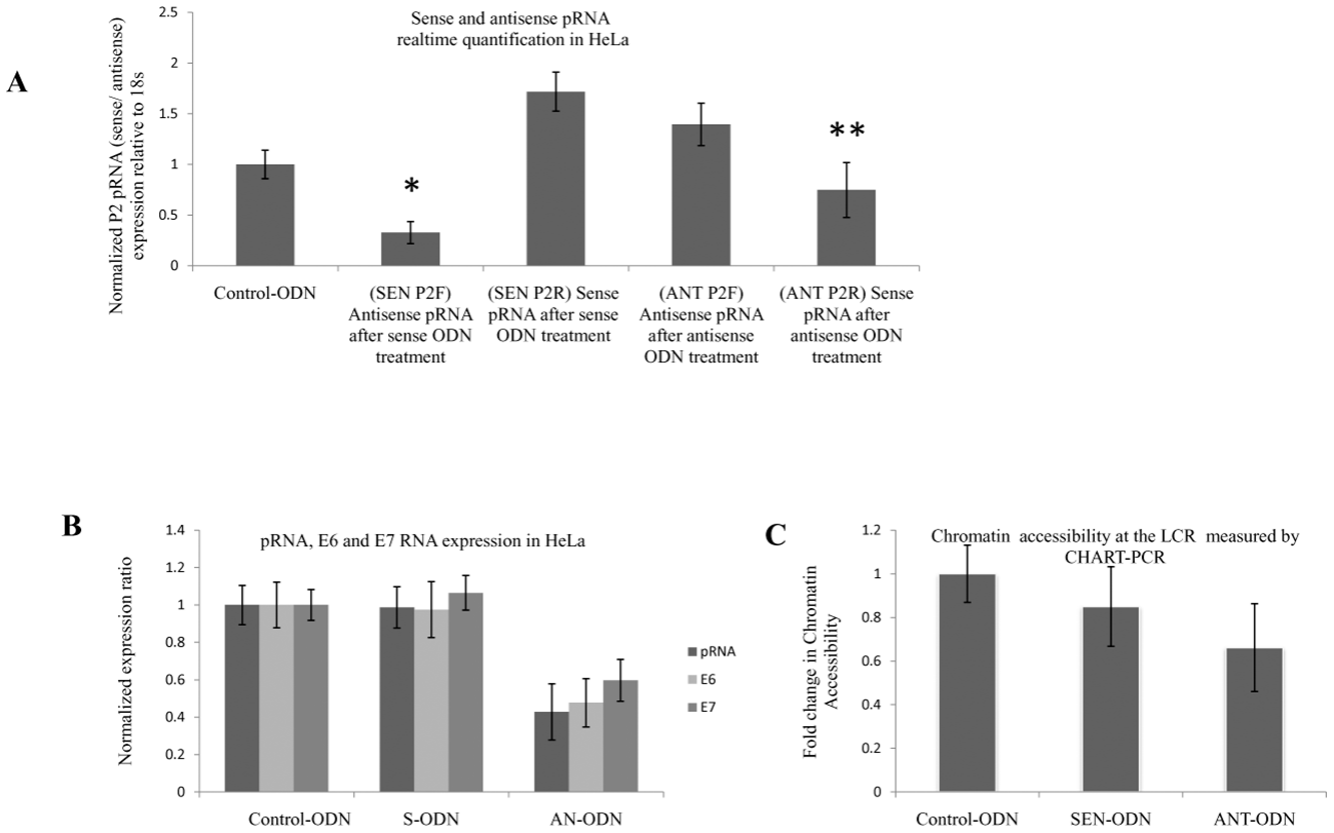
Requirement of pRNA transcription for gene expression. (A) Specificity of ODN targeting:ODNs were checked for modulating bidirectional transcription in HeLa. HeLa cells were treated with respective ODNs followed by analysis of pRNA expression using directional qRT-PCR with P2 primer. SEN P2F: HeLa cells transfected with sense ODN and P2 forward primer (antisense pRNA specific) used in directional RT-PCR SEN P2R: HeLa cells transfected with sense ODN and P2 reverse primer (sense pRNA specific) used in directional RT-PCR, ANT P2F: HeLa cells transfected with antisense ODN and P2 forward primer (antisense pRNA specific) used in directional RT-PCR, ANT P2R: HeLa cells transfected with sense ODN and P2 reverse primer (sense pRNA specific) used in directional RT-PCR Control-ODN: Control ODN treated cells. Expression ratio was calculated with respect to 18S rRNA by REST software normalized to Control dsRNA treated samples. (B) Effect of sense or antisense pRNA knockdown on pRNA, E6 and E7 expression by ODN in HeLa. Control-ODN: Control ODN transfected cells, SEN-ODN: sense ODN transfected cells, ANT-ODN: antisense ODN transfected cells. (*) P < 0.01, (**) P < 0.05. C: Sense pRNA degradation mediated heterochromatization. Significant reduction in MNase digestion 72 hours after antisense ODN transfection while Control or sense ODN transfection had no effect in HeLa cells. Control-ODN: Control ODN transfected cells, SEN-ODN: cells transfected with sense ODN, ANT-ODN: cells transfected with antisense ODN, (*) P < 0.05.

HeLa cells were transfected with respective ODNs (100 nM) and the level of bidirectional pRNA was analyzed by directional qRT-PCRafter three days(Fig 9A). Blocking of sense pRNA by antisense ODN was associated with an induction of antisense pRNA whereas sense ODN mediated antisense pRNA downregulation was associated with an increased level of sense pRNA. Similar results were obtained on treating C-4 II cells with respective ODNs (Figure A in S4 File). We also investigated the expression of pRNA, E6 and E7 upon ODN treatment by real-time PCR in both HeLa and C-4 II cell lines (Fig 9B and Figure B in S4 File) and observed that all target RNAs were decreased (p < 0.02) on treatment with antisense ODN 72 hours post transfection while sense ODN and control ODN treatment had no effect on the expression of pRNA or oncogenes.

Moreover in HeLa cells, MNase accessibility of the target region reduced (40%) significantly upon sense pRNA knockdown by antisense ODN treatment while antisense pRNA degradation by sense ODN had no effect on MNase accessibility in comparison to control ODN treated cells (Fig 9C).

**Fig 10:**
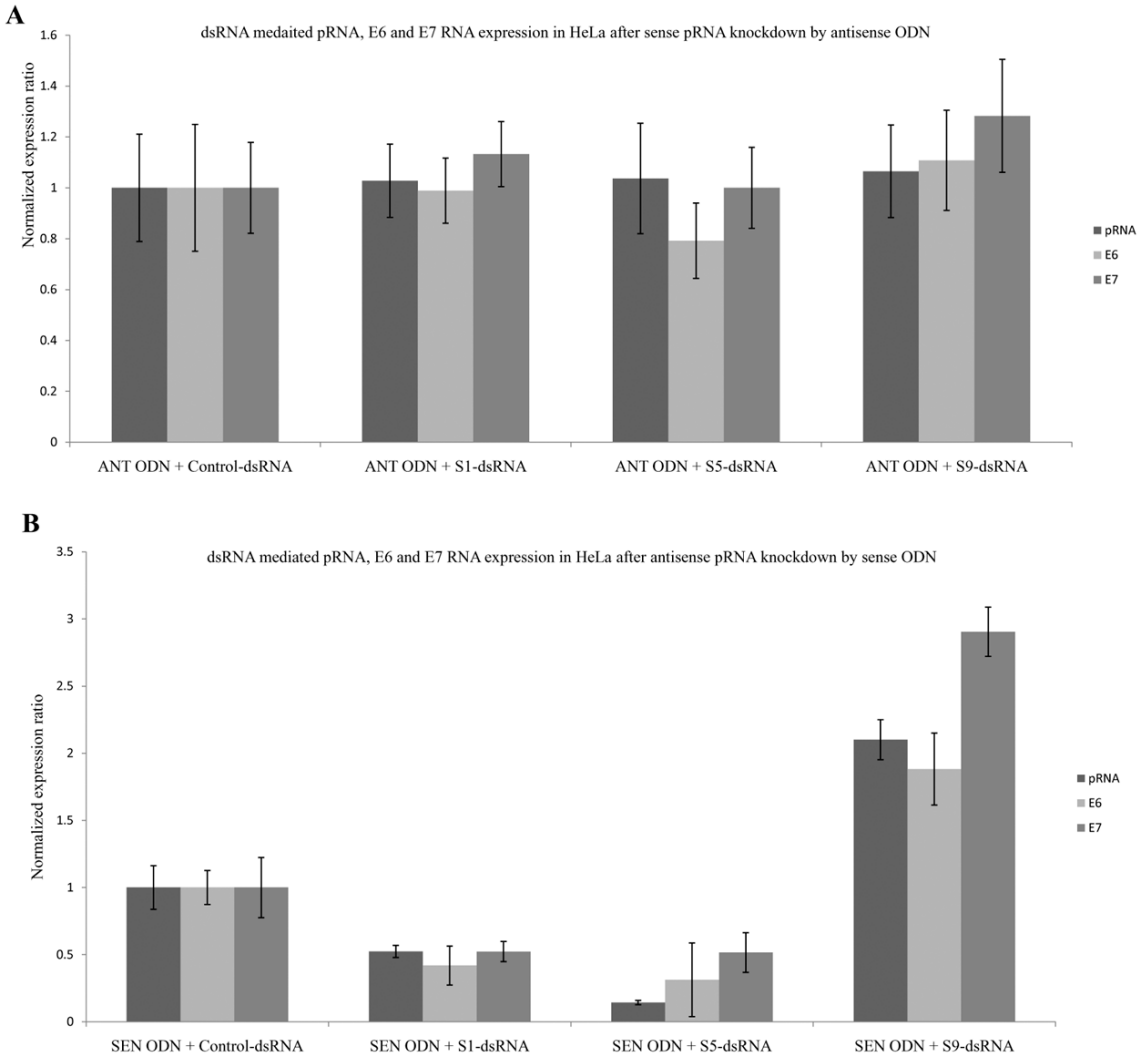
Effect of sense and antisense pRNA downregulation on dsRNA mediated TGS and TGA. HeLa cells were transfected with sense or antisense ODN and 24 hours later transfected with respective dsRNAs. RNA was isolated from the cells and analyzed by Real-Time PCR. Expression ratio was calculated with respect to 18S, POLR2A, PPIA and β-actin by REST software. (A) Hela cells transfected with antisense ODN. (B) HeLa cells transfected with sense ODN. Control-dsRNA: Control dsRNA transfected cells, S1-dsRNA: S1 dsRNA transfected cells, S5-dsRNA: S5 dsRNA transfected cells, S9-dsRNA: S9 dsRNA transfected cells.

We also determined the ability of dsRNAs to modulate oncogene and pRNA expression in the background of sense or antisense pRNA degradation by ODNs. HeLa cells were treated with either sense or antisense ODN and 24 hours later transfected with S1, S5 or S9 dsRNA. RNA was isolated from the transfected cells 72 hours post dsRNA transfection and analyzed for pRNA, E6 and E7 expression. There was abrogation of TGS and TGA upon sense pRNA knockdown (p > 0.05) by ODNs while antisense pRNA knockdown did not affect either TGS or TGA (p < 0.01) (Fig 10A and 10B).

## Discussion

Bidirectional pRNAs of variable sizes were identified in HPV18 positive cell lines HeLa, C-4 I and C-4 II (Fig 1A–1D). The difference in the RT-PCR profile among different cell lines could be due to difference in the site of integration in different cell lines. The epigenetic status like DNA/histone methylation could be responsible for the difference in their transcription. Although there was variation in the size of pRNA across different HPV18 cell lines, pRNAs were present in both sense and antisense orientations at the P2 region of LCR in all the three cell lines (Fig 2, Figures A-B in S1 File). This localization of bidirectional transcription to a particular region of LCR points to a regulatory function of that region and/or of pRNA at that region.

To gain insight into the functional nature of pRNA in regulating E6 and E7 gene expression, we screened multiple dsRNAs against pRNA at this region. Two dsRNAs S1 and S5 designed from two different regions of LCR significantly reduced pRNA expression in C-4 I, C-4 II cell and HeLa cells 72 hours post single transfection (Fig 5A, Figures A-B in S2 File). Downregulation of pRNA by dsRNAs was accompanied by a fall in E6 and E7 oncogene expression in all the three cell lines HeLa, C-4 I and C-4 II (Fig 4B–4D). This signifies that HPV18 LCR associated pRNAs are regulators of E6 and E7 expression.

Earlier findings of TGS suggest that a sense pRNA is the direct target of promoter targeting dsRNAs while antisense pRNA acts as the target of dsRNAs involved in TGA[16,17]. We found that E6 and E7 downregulation was associated with a fall in sense pRNA and a concomitant induction in the antisense pRNA in all cell lines HeLa and C-4 II (Fig 6 and S1 Fig). This suggests that sense pRNA acts as the target of dsRNAs and oncogene downregulation is achieved due to reduction in sense pRNA. These observations support earlier findings which have shown that a sense pRNA is the direct target of promoter targeting dsRNAs[16,17].

Further confirmation for sense pRNA dependent E6 and E7 oncogene expression was obtained by transfecting dsRNAs against non CpG target sites of pRNA. After screening, we identified a dsRNA (S9) that led to an upregulation of pRNA, E6 and E7 in HeLa cells. Apart from E6 and E7 induction by S9 dsRNA transfection, it was also observed that there was a two fold increase in the expression of sense pRNA and a concomitant decrease (by half) in the level of antisense pRNA upon S9 dsRNA transfection (Fig 7A and 7B). These observations further confirm the role of sense pRNA as an important regulator of HPV18 gene regulation i.e. gene expression is dependent on the level of sense pRNA. This finding is consistent with earlier reports of TGA mediated by non CpG region targeting dsRNAs[19].

pRNA mediated TGS and TGA were associated with changing epigenetic modifications in the LCR. S1 and S5 dsRNAs decreased target MNase accessibility (by 55% and 70% respectively) in comparison to control dsRNA treated cells while S9 transfection led to euchromatization of the target region (Fig 8A). The mechanism involved in chromatin remodeling post dsRNA mediated sense pRNA modulation was elucidated by ChIP assay for histone methylation marks. S1 and S5 transfection in HeLa cells led to increase in repressive methylation marks H3K9me2 and H3K27me3 (Fig 8Bi and 8Bii). In addition, HDAC inhibitor TSA led to loss of S1 and S5 mediated TGS indicating that TSA reverted back S1 and S5 mediated heterochromatization. These two observations signify that TGS associated heterochromatization of target region is brought about by increased methylation and deacetylation of target promoter. S9 dsRNA mediated TGA showed no change in the enrichment of H3K27me3 and remained unaffected with TSA treatment but activating histone marker H3K4me2 increased 5 fold at the target region (Fig 8Biii, 8Biv and 8Cii). This implies that S9 mediated euchromatization is brought about by increased activating histone methylation at the target DNA region.

S1 and S5 dsRNA mediated TGS was not associated with CpG methylation of the target or the adjacent DNA region as found by bisulphite sequencing and AZA treatment (inhibitor of DNA methyltransferases) (Figures A-F in S3 File). AZA treatment had no effect on dsRNA mediated TGS in HeLa cells. This is in accordance with various previous studies of TGS mediated even in the absence of DNA methylation[24].

We also ascertained the direct function of sense and antisense pRNAs in the regulation of gene expression utilizing ODNs. Blocking sense pRNA by antisense ODN was associated with an induction of antisense pRNA whereas sense ODN mediated antisense pRNA downregulation was associated with an activation of sense pRNA (Fig 9A). In addition, while blocking of sense pRNA by ODNs knocked down the expression of pRNA, E6 and E7; there was no effect on the expression of pRNA or oncogenes after antisense pRNA knockdown by sense ODN. This further confirms our earlier observation about sense pRNA dependent expression of E6 and E7. Although sense pRNA knockdown by antisense ODN led to both decrease in oncogene expression and MNase accessibility (Fig 9C), the reduction was low when compared to dsRNA induced silencing. This suggests that the downregulation of sense pRNA is not sufficient to cause TGS by itself, optimal silencing is achieved after dsRNA-pRNA interaction and consequent recruitment of various factors to the target site, thus supporting the RNA-RNA model of TGS[16]. Similarly TGA is brought about by induction of sense pRNA and expression of sense pRNA is absolutely required for dsRNA induced TGA. dsRNA-pRNA interaction mediated recruitment of various activating protein factors to the target site is probably required for effective TGA.

Both TGS and TGA were lost after sense pRNA knockdown while these processes remained unaffected after antisense pRNA knockdown (Fig 10A and 10B). This implies that sense pRNA plays a pivotal role in dsRNA induced gene silencing and activation and is required for both the processes to take place.

We have thus identified and demonstrated the role of HPV18 LCR associated RNAs in regulating E6 and E7 oncogene expression in cervical cancer cells. dsRNA mediated silencing and activation of pRNA demonstrated that the expression of downstream oncogenes was directly linked to the expression of a sense oriented pRNA suggesting its possible role as a natural regulator of gene expression in integrated HPV18. This newly identified promoter associated transcript helps us in further understanding of HPV18 gene regulation and the identified dsRNAs against the pRNAs may prove valuable in therapeutic treatment against HPV18 induced cervical cancer. Thus this finding presents a novel mechanism of gene regulation mediated by promoter associated transcripts in HPV18 and may possibly have a bearing in the transcription of other genes as well.

## Supporting Information

S1 Fig Change in bidirectional pRNA transcription in C-4 II cells after S1 or S5 dsRNA transfection. Sense and antisense pRNA expression in C-4 II cells.
(TIF)

S1 File Determination of pRNA orientation in HPV18 integrated C-4 and C-4 II cells. (A) Orientation of pRNA at P2 region of LCR in C-4 I cells. (B) Orientation of pRNA at P2 and P4 regions of LCR in C- 4 II cells.
(TIF)

S2 File pRNA expression in C-4 II and C-4 I cells. (A) pRNA expression in C-4 II. (B) pRNA expression in C-4 I.
(TIF)

S3 File DNA methylation has no role in TGS or TGA. (A) Chromatogram sequence obtained after dsRNA transfection followed by bisulphite treatment in HeLa cells. The CpG sites in the target region have been underlined. All the Cytosines in the target region were Controlverted to Thymines indicating thate there was no methylation at these sites. A&D: Control dsRNA transfection, B: S5 dsRNA transfection, C: S9 dsRNA transfection, E: S1 tranfection. (F)DNA methyltransferase inhibition has no role in TGS. Expression ratio of pRNA, E6 and E7 after S1 and S5 transfection. HeLa cells were transfected with respective dsRNAs and 24 hours later treated with DMSO (Fig 9B) or AZA followed by RNA isolation 48 hours after the treatment. Control: Control dsRNA treated cells, S1: S1 dsRNA transfected cells, S5: S5 dsRNA transfected cells.
(TIF)

S4 File Requirement of pRNA transcription for gene expression. (A) Specificity of ODN targeting C-4 II cells.(B) Effect of sense or antisense pRNA knockdown by ODN in C-4 II cells.
(TIF)

S1 Table Name and sequence of dsRNAs and ODNs used in this study. All the dsRNA have a TT overhang at the 3' end.
(DOCX)

S2 Table List of primers and oligonucleotides used. Name and sequence of the primers used in PCR and RT-PCR in this study.
(DOCX)

S3 Table Primers used for Bisulphite sequencing. Name and sequence of primers used in Bisulphite sequencing PCR.
(DOCX)
