# Supplementary figures and images for: Gene Silencing and Activation of Human Papillomavirus 18 Is Modulated by Sense Promoter Associated RNA in Bidirectionally Transcribed Long Control Region
Source: PLoS One. 2015 Jun 5;10(6):e0128416. doi: 10.1371/journal.pone.0128416 (PMC4457724; doi:10.1371/journal.pone.0128416)

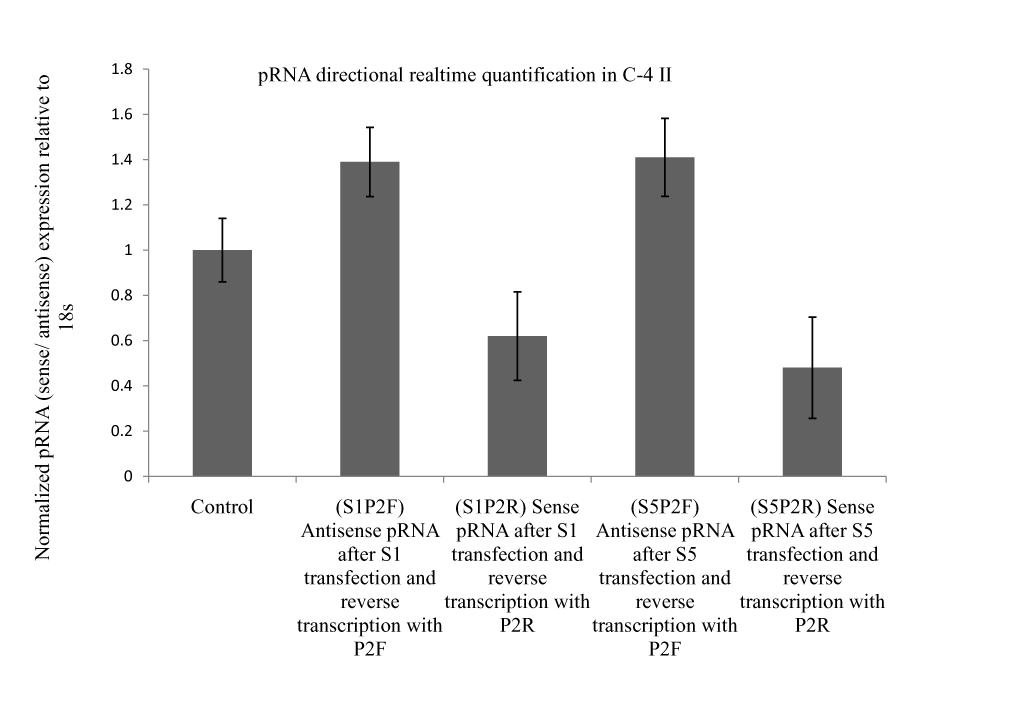

Supplement: S1 Fig — Sense and antisense pRNA expression in C-4 II cells. (TIF) [file pone.0128416.s001.tif]

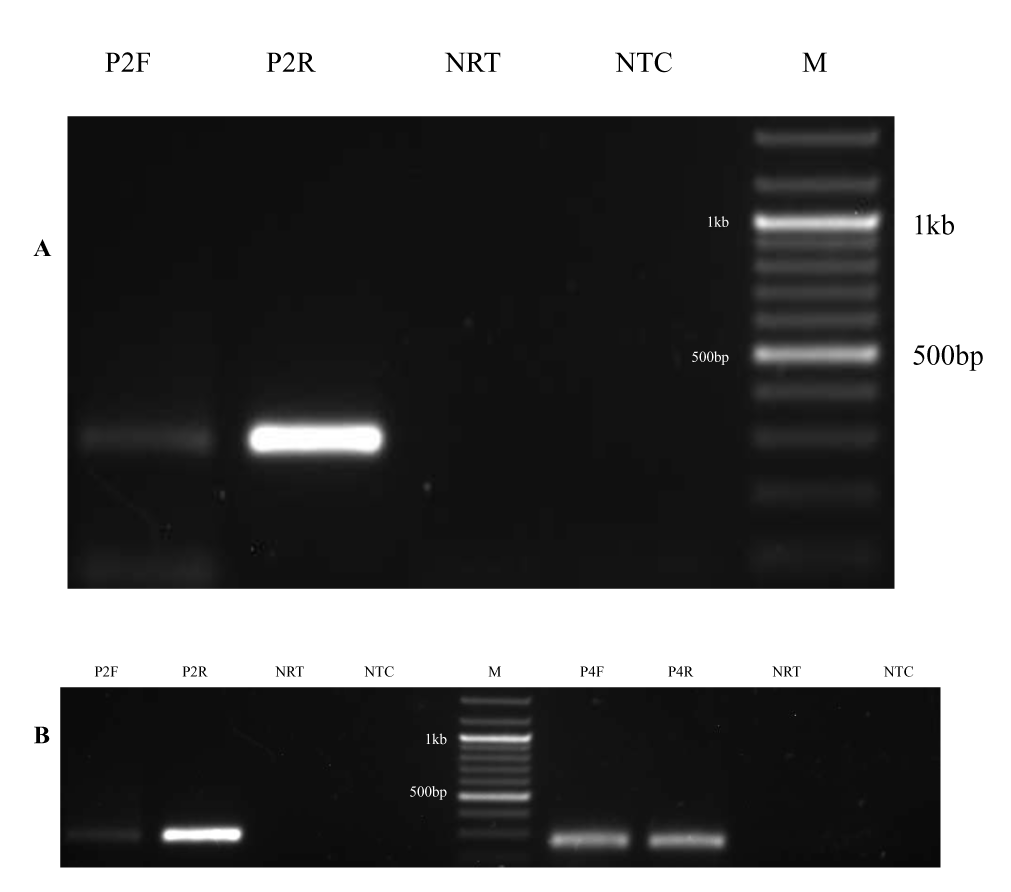

Supplement: S1 File — (A) Orientation of pRNA at P2 region of LCR in C-4 I cells. (B) Orientation of pRNA at P2 and P4 regions of LCR in C- 4 II cells. (TIF) [file pone.0128416.s002.tif]

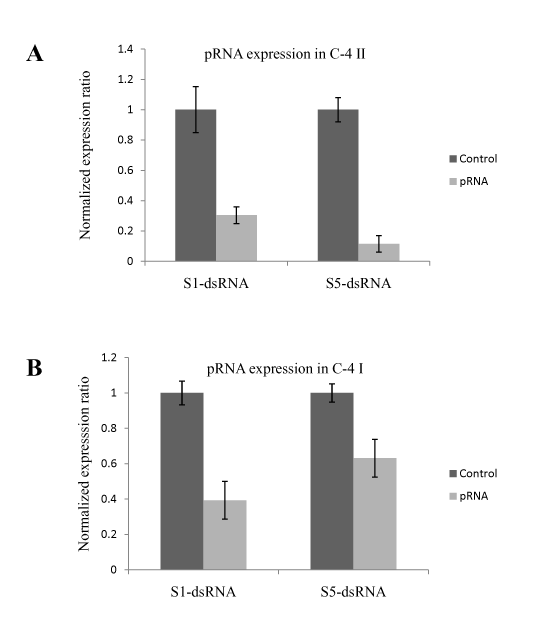

Supplement: S2 File — (A) pRNA expression in C-4 II. (B) pRNA expression in C-4 I. (TIF) [file pone.0128416.s003.tif]

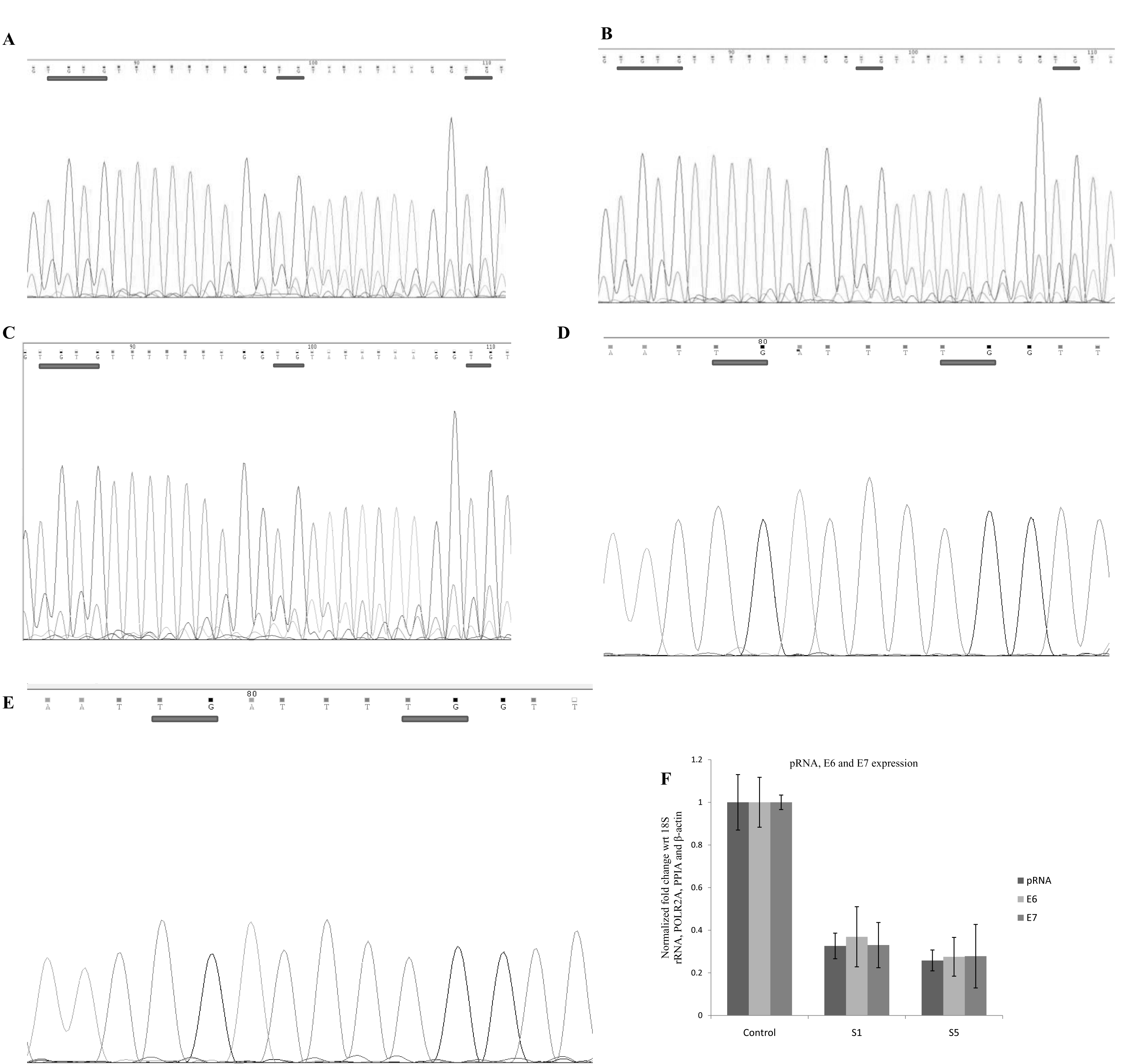

Supplement: S3 File — (A) Chromatogram sequence obtained after dsRNA transfection followed by bisulphite treatment in HeLa cells. The CpG sites in the target region have been underlined. All the Cytosines in the target region were Controlverted to Thymines indicating thate there was no methylation at these sites. A&D: Control dsRNA transfection, B: S5 dsRNA transfection, C: S9 dsRNA transfection, E: S1 tranfection. (F)DNA methyltransferase inhibition has no role in TGS. Expression ratio of pRNA, E6 and E7 after S1 and S5 transfection. HeLa cells were transfected with respective dsRNAs and 24 hours later treated with DMSO (Fig 9B) or AZA followed by RNA isolation 48 hours after the treatment. Control: Control dsRNA treated cells, S1: S1 dsRNA transfected cells, S5: S5 dsRNA transfected cells. (TIF) [file pone.0128416.s004.tif]

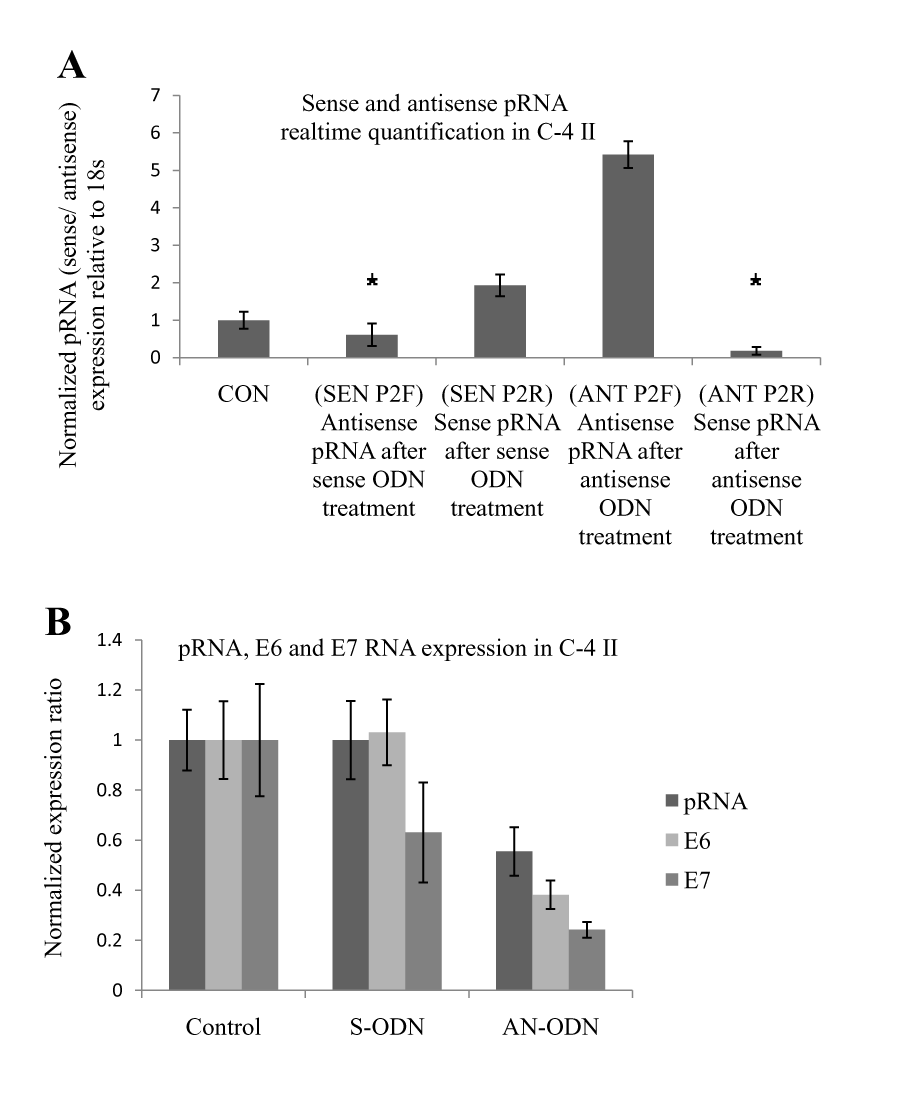

Supplement: S4 File — (A) Specificity of ODN targeting C-4 II cells.(B) Effect of sense or antisense pRNA knockdown by ODN in C-4 II cells. (TIF) [file pone.0128416.s005.tif]
