# Supplementary material for: Gene Silencing and Activation of Human Papillomavirus 18 Is Modulated by Sense Promoter Associated RNA in Bidirectionally Transcribed Long Control Region
Source: PLoS One. 2015 Jun 5;10(6):e0128416. doi: 10.1371/journal.pone.0128416 (PMC4457724; doi:10.1371/journal.pone.0128416)
Supplement: S1 Table — All the dsRNA have a TT overhang at the 3' end. (DOCX) [file pone.0128416.s006.docx]

| Name of dsRNA/ODN | Sequence 5' to 3' |
| --- | --- |
| dsRNA S1 sense | UGCUGUGCAACCGAUUUCGTT |
| dsRNA S1 antisense | CGAAAUCGGUUGCACAGCATT |
| dsRNA S2 sense | CUGUGCAACCGAUUUCGGUTT |
| dsRNA S2 antisense | ACCGAAAUCGGUUGCACAGTT |
| dsRNA S3 sense | GUGCAACCGAUUUCGGUUGTT |
| dsRNA S3 antisense | CAACCGAAAUCGGUUGCACTT |
| dsRNA S4 sense | AUUGGCGCGCCUCUUUGGCTT |
| dsRNA S4 antisense | GCCAAAGAGGCGCGCCAAUTT |
| dsRNA S5 sense | UGGCGCGCCUCUUUGGCGCTT |
| dsRNA S5 antisense | GCGCCAAAGAGGCGCGCCATT |
| dsRNA S6 sense | GCGCGCCUCUUUGGCGCAUTT |
| dsRNA S6 antisense | AUGCGCCAAAGAGGCGCGCTT |
| dsRNA S7 sense | GCGCCUCUUUGGCGCAUAUTT |
| dsRNA S7 antisense | AUAUGCGCCAAAGAGGCGCTT |
| dsRNA S8 sense | CACCUGGUAUUAGUCAUUUTT |
| dsRNA S8 antisense | AAAUGACUAAUACCAGGUGTT |
| dsRNA S9 sense | CCUGGUAUUAGUCAUUUUCTT |
| dsRNA S9 antisense | GAAAAUGACUAAUACCAGGTT |
| dsRNA S10 sense | UGGUAUUAGUCAUUUUCCUTT |
| dsRNA S10 antisense | AGGAAAAUGACUAAUACCATT |
| dsRNA control sense | GUUCUCUGGUAUAUGAUCUTT |
| dsRNA control antisense | AGAUCAUAUACCAGAGAACTT |
| Sense ODN | C*T*T*T*G*G*C*T*T*A*T*G*T*C*T*G*T*G*G*T*T |
| Antisense ODN | A*A*C*C*A*C*A*G*A*C*A*T*A*A*G*C*C*A*A*A*G |
| Control ODN | C*A*C*A*A*G*A*T*G*C*C*C*T*C*T*G*G*G*C*T*T |
