# Supplementary material for: Gene Silencing and Activation of Human Papillomavirus 18 Is Modulated by Sense Promoter Associated RNA in Bidirectionally Transcribed Long Control Region
Source: PLoS One. 2015 Jun 5;10(6):e0128416. doi: 10.1371/journal.pone.0128416 (PMC4457724; doi:10.1371/journal.pone.0128416)
Supplement: S2 Table — Name and sequence of the primers used in PCR and RT-PCR in this study. (DOCX) [file pone.0128416.s007.docx]

| Prmier ID | Sequence 5′ to 3′ |
| --- | --- |
| P1 Forward | TTGTGTTTGTATGTCCTGTGTTTGTG |
| P1 Reverse | TATATAGCCCAACAAGCAACACC |
| P2 Forward used also in ChIP and MNase assay | TTGTGGTATGGGTGTTGCTTGTT |
| P2 Reverse used also in ChIP and MNase assay | CACCTGGACAGGAAAATGACTAAT |
| P3 Forward | CGCCTCTTTGGCGCATATAA |
| P3 Reverse | AGGGTAGACAGAATGTTGGACATGA |
| P4 Forward | AAGCTAATTGCATACTTGGCTTGTA |
| P4 Reverse | TTGTGGTGTGTTTCTCACATCTTTT |
| HPV18 E6 Forward | GCGACCCTACAAGCTACCTG |
| HPV18 E6 Reverse | TGCAGCATGGGGTATACTGT |
| HPV18 E7 Forward | ACCTAAGGCAACATTGCAAG |
| HPV18 E7 Reverse | CTGAACACCCTGTCCTTTGT |
| L1 Forward | TGC TGC ACC GGC TGA AAA TAA |
| P2F2 | GGTATGGGTGTTGCTTGTTGGGCTA |
| P2For2 | GTTTGTGGTATGGGTGTTGCT |
| P2F nested | CCTCCATTTTGCTGTGCAACC |
| P2RN | GCGCCTTATATGCGCCAAAG |
| 18S Forward | GTAACCCGTTGAACCCCATT |
| 18S Reverse | CCATCCAATCGGTAGTAGCG |
| PPIA Forward | CACCGTGTTCTTCGACATTG |
| PPIA Reverse | TTCTGCTGTCTTTGGGACCT |
| Actin Forward | AGAAAATCTGGCACCACACC |
| Actin Reverse | TAGCACAGCCTGGATAGCAA |
| POLR2A Forward | CATCAAGAGAGTCCAGTTCGG |
| POLR2A Reverse | CCCTCAGTCGTCTCTGGGTA |
| Chr16 Forward | GTCTCTTTCTTGTTTTTAAGCTGGG |
| Chr16 Reverse | TGAGCTCATTGAGACATTTGG |
| HPV16 E6 Forward | AGCGACCCAGAAAGTTACCA |
| HPV16 E6 Reverse | GCATAAATCCCGAAAAGCAA |
| HPV16 E7 Forward | ACAAGCAGAACCGGACAGAG |
| HPV16 E7 Reverse | GCCCATTAACAGGTCTTCCA |
| OAS1 Forward | TTCTCCACCTGCTTCACAGA |
| OAS1 Reverse | GAGCTCCAGGGCATACTGAG |
