# Supplementary material for: Gene Silencing and Activation of Human Papillomavirus 18 Is Modulated by Sense Promoter Associated RNA in Bidirectionally Transcribed Long Control Region
Source: PLoS One. 2015 Jun 5;10(6):e0128416. doi: 10.1371/journal.pone.0128416 (PMC4457724; doi:10.1371/journal.pone.0128416)
Supplement: S3 Table — Name and sequence of primers used in Bisulphite sequencing PCR. (DOCX) [file pone.0128416.s008.docx]

| Prmier ID | Sequence 5′ to 3′ |
| --- | --- |
| Bisulfite outer Forward | TGTTGTTTGTTGGGTTATAT |
| Bisulfite outer Reverse | CAATTAACTTAAATAAAAAC |
| Bisulfite inner Forward S1 | GTTTGTTGGGTTATATATTG |
| Bisulfite inner Reverse S1 | AATATATAAAAACAATACCC |
| Bisulfite inner Forward S5/S9 | TTGTTTTTGGTTTATGTTTGTGG |
| Bisulfite inner Reverse S5/S9 | ACAATTTTATTACTTAAAAA |
